# Improving Trainee Experience of Raising Concerns: Redefining a Representative Structure for Post-Graduate Doctors in Training

**DOI:** 10.1192/bjo.2024.382

**Published:** 2024-08-01

**Authors:** Katherine Hubbard, Sian Davies, Vicki Ibbett, Shay-Anne Pantall, Ruth Scally

**Affiliations:** Birmingham and Solihull Mental Health NHS Foundation Trust, Birmingham, United Kingdom

## Abstract

**Aims:**

As part of a wider quality improvement project (QIP) aiming to improve trainees’ experiences with ‘Raising Concerns’ in a large mental health trust, we sought to improve the trainee representative (rep) structure. This would give trainees more transparent processes and provide intermediaries by which to raise concerns. Based on change ideas generated from our driver diagram, roles were created to coordinate meetings and represent specific groups of trainees and on-call rotas.

**Methods:**

Prior to August 2022, there were an undefined number of ‘Senior House Officer’ (SHO) reps who were recruited informally by the Post-Graduate Medical Education Team. The duties of these reps were not clearly detailed. As part of our first ‘Plan, Do, Study, Act’ (PDSA) cycle, we identified groups of trainees that needed additional representation (International Medical Graduates [IMGs], Less than Full Time trainees [LTFT]) and introduced a Wellbeing Rep to cover all training grades. Specifically for SHOs, we introduced three core roles (Rota/Placement, Inclusion, and Social) and individual roles for the six on-call rotas. Following the implementation of this rep structure, we gathered quantitative data, including whether trainees had utilised the reps and how effective they were in raising concerns, and qualitative feedback. We gathered data from both the reps and the whole cohort of trainees. We then started another PDSA cycle in August 2023.

**Results:**

On a 1–5 scale (5 = very effective), the average response from trainees for how effective the trust reps were in supporting raising concerns was 3.8 (5 responders), with no trainees who responded feeling that any of the rep roles needed restructuring. However, the rep survey highlighted that the following roles needed restructuring: Rota/Placement rep, Social rep, and Rota reps. The Rota/Placement role was highlighted as being unnecessary due to the existence of individual rota reps, but there was a need for a ‘lead’ rep to coordinate rep meetings and induction. Unfortunately, a Social rep was not recruited, however it was identified that due to the importance of the role more than one trainee may be required to arrange social events.

**Conclusion:**

Overall, the trainee response to the new rep structure has been neutral/effective, but we hope to obtain more responses in the next PDSA cycle. The rep feedback highlighted the need for coordinator roles to improve cohesion. The results have informed change ideas which we implemented in August 2023. The second PDSA cycle will be completed in July 2024.

